# A comparison of the *Giardia lamblia *trophozoite and cyst transcriptome using microarrays

**DOI:** 10.1186/1471-2180-11-91

**Published:** 2011-05-04

**Authors:** Zahra Faghiri, Giovanni Widmer

**Affiliations:** 1 Division of Infectious Diseases, Tufts Cummings School of Veterinary Medicine, 200 Westboro Road, North Grafton, MA, USA

## Abstract

**Background:**

Compared with many protists, *Giardia lamblia *has a simple life cycle alternating between cyst and trophozoite. Most research on the molecular biology of *Giardia *parasites has focused on trophozoites and the processes of excystation and encystation, whereas cysts have attracted less interest. The striking morphological differences between the dormant cyst and the rapidly dividing and motile trophozoite implies profound changes in the metabolism as the parasite encysts in the host's intestine and excysts upon ingestion by a new host.

**Results:**

To investigate the magnitude of the transcriptional changes occurring during the *G. lamblia *life cycle we compared the transcriptome of *G. lamblia *trophozoites and cysts using single-color oligonucleotide microarrays. Cysts were found to possess a much smaller transcriptome, both in terms of mRNA diversity and abundance. Genes encoding proteins related to ribosomal functions are highly over-represented. The comparison of the transcriptome of cysts generated in culture or extracted from feces revealed little overlap, raising the possibility of significant biological differences between the two types of cysts.

**Conclusions:**

The comparison of the *G. lamblia *cyst and trophozoite transcriptome showed that transcripts of most genes are present at a lower level in cysts. This global view of the cyst and trophozoite transcriptome complements studies focused on the expression of selected genes during trophozoite multiplication, encystation and excystation.

## Background

*Giardia lamblia *(*G. duodenalis, G. intestinalis*) is a diplomonad parasite which causes over 20,000 reported cases of giardiasis a year in the United States [1]. In addition to its importance as a widespread human and animal pathogen, the long evolutionary history of the diplomonad lineage makes *G. lamblia *an interesting system for studying eukaryotic evolution and the evolution of parasitism. Research on *G. lamblia *is aided by the fact that the entire life cycle can be studied outside the host, and that the differentiation from cyst to trophozoite and the reverse process of encystation can be reproduced in vitro. Recently, the availability of the complete genome sequence [2-5] have facilitated genome-wide analyses.

Although many *Giardia *proteins and organelles have been studied in detail, genome-wide studies of the transcriptome and proteome have been few [6-11]. No microarray analyses of the transcriptome of cysts obtained from infected animals have to our knowledge been performed. Serial Analysis of Gene Expression (SAGE) was used to survey changes in the *G. lamblia *transcriptome during encystation and excystation [9]. This study grouped about 10% of predicted *G. lamblia *genes into six clusters with related transcriptional profile. SAGE was also used to analyze the relative abundance of transcripts encoding cytoskeleton proteins [8]. This analysis found that the level of mRNA transcripts encoding proteins localized in the adhesive decreases as the parasite encysts, and also found a lack of association between mRNA and protein level. Morf and co-workers focused on transcriptional changes associated with encystation [12]. This study used micoarrays to identify genes which are induced during encystation and found evidence of transcriptional co-regulation mediated by a shared transcription factor binding motif in the promoter region of such genes.

The extensive morphological changes which take place during the parasite's life cycle have for years motivated the study of transcriptional regulation of selected genes during differentiation. Reverse-transcription PCR has been frequently used to monitor changes in the level of specific mRNA transcripts, such as those encoding enzymes involved in energy metabolism [13], recombination [14], structural functions [15] or regulatory functions [16].

We wished to compare on a global level the transcriptional landscape of trophozoites and cysts. We found that in cysts many genes are either not transcribed, or that the transcripts they encode are too rare to be detected with microarrays.

## Results

### Analysis of the cyst transcriptome

**Figure 1:**
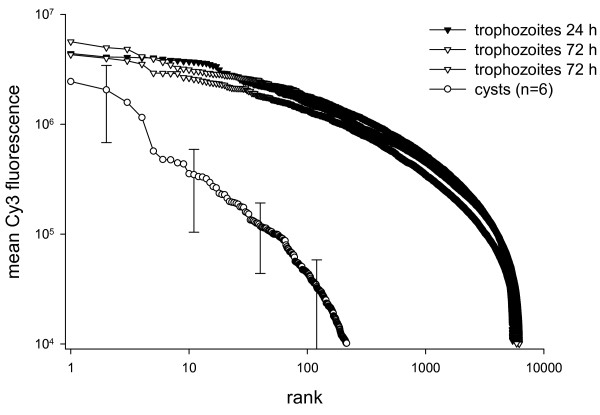
**Comparison of cyst and trophozoite transcriptome**. Cy3 fluorescence from two replicate trophozoite microarray hybridizations and mean fluorescence from six cyst microarrays are ranked in order of decreasing fluorescence intensity. Illustrating the difference in mRNA abundance between life cycle stages 5454 and 6198 trophozoite genes, respectively, exceeded 10,000 fluorescence units, but only 215 cyst genes were above this threshold. Because fluorescence values are ranked, vertically aligned data point do not necessarily originate from the same gene. Error bars show standard deviation for the six cyst replicates. Tropohozoite datapoints are means of two replicate spots. All replicates are biologically independent. Both variables are plotted on a log scale. Trophozoites (isolate GS) and cysts (isolate H3) of assemblage B were used in this comparison.

The cyst and trophozoite transcriptome were compared by plotting mean Cy3 fluorescence values from six replicate microarrays hybridized with cDNA from independent live cyst suspensions and two replicate microarrays hybridized with trophozoite cDNA. The two trophozoites samples originated from a culture of assemblage B GS isolate in exponential phase of growth harvested 24 h post-inoculation and from a stationary culture harvested at 72 h. Cysts of assemblage B isolate H3 were obtained from experimentally infected gerbils. Their viability estimated by propidium iodide exclusion [17] ranged from 90% to 93% in three randomly selected cyst samples. In Figure 1 mean cyst and trophozoite Cy3 fluorescence values are ranked from in order of decreasing intensity. For this analysis only Cy3 data were used. Of 6913 genes represented on the *G. lamblia *microarray, 5454 and 6189 transcripts, respectively, were detected in trophozoites. These numbers include fluorescence values exceeding a threshold of 10,000 fluorescent units. This limit was set based on background fluorescence emitted by empty microarray positions, which averaged 1713 Cy3 fluorescence units (n = 4650). In contrast, only 215 transcripts were detected in cysts, equivalent to 3% of 6913 genes. Although each of the 2 trophozoite and 6 cyst datasets originated from different microarrays, the data are comparable because each microarray was hybridized with a standardized amount of cDNA probe synthesized from the same amount total RNA. The error bars in Figure 1 clearly show that the differences between cysts and trophozoites exceed the variability among biological replicates. This analysis thus demonstrates that for equal amount of total RNA trophozoites synthesize more mRNA and that the mRNA transcriptome is more diverse than in cysts.

Although the cyst and trophozoite transcriptome compared in these experiments both belonged to assemblage B, we investigated whether sequence polymorphism between the assemblage A sequence on which the *G. lamblia *microarray is based and assemblage B probe could reduce hybridization. Using the same single-color experimental design, we compared fluorescence values for microarrays hybridized with cDNA from assemblage A and B trophozoites (Additional file 1). Means of Cy3 fluorescence over all *G. lamblia *spots on the array for the assemblage B probe was 3.0 × 10^5^, 2.2 × 10^5^, and 2.9 × 10^5 ^fluorescence units, whereas for assemblage A probe mean fluorescence of 0.9 × 10^5^, 1.5 × 10^5 ^and 3.2 × 10^5 ^were obtained. Thus, the fact that probe and array are derived from different assemblages does not influence the results. These results are consistent with the interpretation of Figure 1.

Further insight into the cyst transcriptome was gained by identifying enriched gene ontology (GO) terms among the 215 genes which generated the highest fluorescence values in cysts (Additional file 2). The list of highly expressed cyst genes was significantly enriched for the molecular function "structural constituents of ribosomes" (p = 3.15 × 10^-28^), as well as other cellular constituents and biological processes related to ribosome (p = 1.03 × 10^-20^) and ribonucleoprotein complex (p = 3.13 × 10^-16^). These three GO categories had the lowest probability values. Similar GO categories were identified among the 215 highest ranking trophozoite transcripts. "Structural constituents of ribosomes" was again the top-ranking molecular function (p = 7.9 × 10^-28^) "ribonucleoprotein complex" (p = 2.9 × 10^-17^) and "non-membrane bound organelle" (p = 1.2 × 10^-11^). In contrast to the overall functional similarity between cyst and trophozoite transcriptome, when considering only genes with the highest mRNA level significant differences were apparent between cyst and trophozoite. In addition to ribosomal proteins, the annotation of the most highly expressed cyst transcripts includes several structural proteins and variant surface proteins (Table 1). Only one gene (ubiquitin) featured in the cyst and trophozoite list of highly expressed genes. These analyses reveal that in spite of the over-representation of ribosomal functions in both stages, the cyst and trophozoite transcriptome are not only quantitatively but also qualitatively different.

**Table 1:** Gene ID and annotation of 14 most expressed cyst and trophozoite genes

cysts		trophozoites	
**gene ID**	**annotation**	**gene ID**	**annotation**

GL50803_7110	ubiquitin	GL50803_16044	hypothetical

GL50803_135002	histone H4	GL50803_10919	ribosomal protein S10B

GL50803_121046	histone H2B	GL50803_17153	α11 giardin

GL50803_9848	dynein light chain	GL50803_31374	hypothetical

GL50803_32146	α-tubulin	GL50803_31532	ribosomal protein L18a

GL50803_135231	histone H3	GL50803_7110	ubiquitin

GL50803_6430	14-3-3 protein	GL50803_15228	ribosomal protein S15A

GL50803_4812	β-giardin	GL50803_116306	variant surface protein

GL50803_16114	ribosomal protein L36-1	GL50803_35316	protein 21.1

GL50803_19182	hypothetical	GL50803_31107	hypothetical

GL50803_15046	ribosomal protein L26	GL50803_135002	histone H4

GL50803_137610	variant surface protein	GL50803_32002	ribosomal protein L10

GL50803_136001	variant surface protein	GL50803_6135*	ribosomal protein S17

GL50803_16501	variant surface protein	GL50803_35621	protein 21.1

### Validation of microarray data

**Figure 2:**
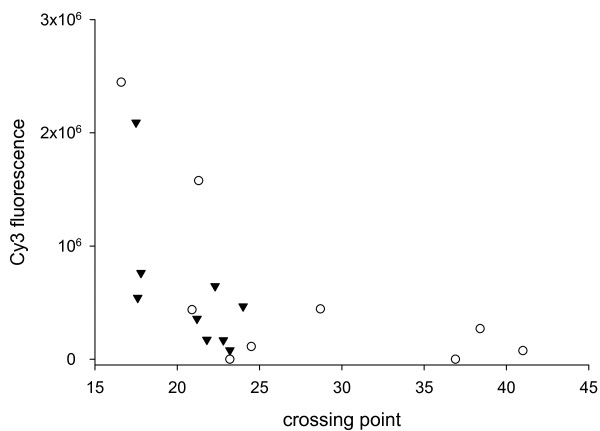
**Validation of microarray data with quantitative PCR**. Mean Cy3 fluorescence was plotted against RT PCR crossing point for live cysts (6 microarrays) and 24-h trophozoites (3 microarrays). The plot shows the expected inverse correlation between the two variables. Crossing Point values shown in Table 3 in columns "Trophozoites 24 h" and "Cysts" were used for the 10 genes listed in the table. Where the same gene was analyzed in replicate PCR analyses the mean of the observed Crossing Points was used. Triangles, trophozoites; circles, cysts.

The abundance of selected transcripts was further investigated with quantitative PCR. Equal portions of cDNA were amplified with primers specific for 10 *G. lamblia *genes (Table 2). The raw Crossing Point values are displayed in Table 3 together with the log_2 _of the cyst/trophozoite ratios. The ratios are generally in agreement with the microarray data presented in Figure 1 in showing negative values for most genes. A plot of Crossing Point against the corresponding microarray fluorescence value is shown in Figure 2. The graph displays the expected inverse correlation, where high Crossing Points correspond to low fluorescence and vice versa. This correlation was found for cyst and trophozoite data.

**Table 2:** PCR primers

Gene annotation	Locus	Sequence*	Annealing temperature
histone H2B	GL50803_121046	F:CGCCTGATGAAGAAGACG R:GTGTTCCGCTTGCTGA	60

14-3-3 protein	GL50803_6430	F:CGGTATGGAAGGCGAGCT R:GCTTGAGGATGTCGTTGC	61

*Giardia *troph antigen GTA-1	GL50803_17090	F:GCCCGTAGAGTTCTGG R:CGTCACTATCTCCCCG	61

ubiquitin	GL50803_7110	F:GTTGAGCCCACAGATACC R:GTTACCACCACGGAGG	61

β-giardin	GL50803_4812	F: ATGTTCACCTCCACCC R: CGGAAGTTTGCAGCCA	62

centrin	GL50803_6744	F: GCAAACCAAACGCTCG R: CCAGACGTATCCACCTC	61

α-tubulin	GL50803_103676	F: CAAGTACATGGCGTGCTGCATGAT R:TAGTTGATGCCGACCTTGAAGCCT	61

SALP-1	GL50803_4410	F: CCGCGCCGACCCCACG R: GCTCATCCAGCATCTTGTCC	61

endothelin-converting enzyme 2	GL50803_4349	F:CATATCACCTTCCTGA R:GACCTGGGAGACATCAATGG	61

mitotic spindle checkpt. MAD2	GL50803_100955	F:GGCTACCCAGACCAAG R:CCCGCCTATCGGAAGA	61

*F, forward primer; R, reverse primer

**Table 3:** Summary of quantitative PCR validation

Gene_ID^§^	annotation	neg contr	troph. 24 h	troph. 72 h	cysts	24 h troph/cysts*
121046	histone H2B^†^		17.8	16.4	18.5	-0.1
					24.1	

6430	14-3-3 prot.	> 41	17.6	15.6	20.9	-0.2

17090	troph antig GTA-1		24.0	22.1	38.4	-0.7

			17.1	14.7	13.8	0.3
7110	Ubiquitin				17.3	
		> 41	17.9		18.8	-0.1

		38.4	21.8		27.5	-0.3
4812	β-giardin	38.2	22.0		29.2	-0.4
		37.3	22.1		29.6	-0.4

15525	centrin	38.2	22.8	23.0	36.9	-0.7

103676	α-tubulin	37.3	21.8	21.9	24.5	-0.2

5347	SLAP-1	37.2	23.2	21.8	23.2	0.0

4349	ECE2	> 41	21.2	20.6	> 41	-1.0

100955	MAD2	> 41	23.3	22.1	38.6	-0.7

^§ ^GL50803 prefix omitted

* log_2_(ratio)

^† ^Crossing points from individual experiments are shown on separate lines

### Comparison of SAGE and microarray cyst transcriptome

**Figure 3:**
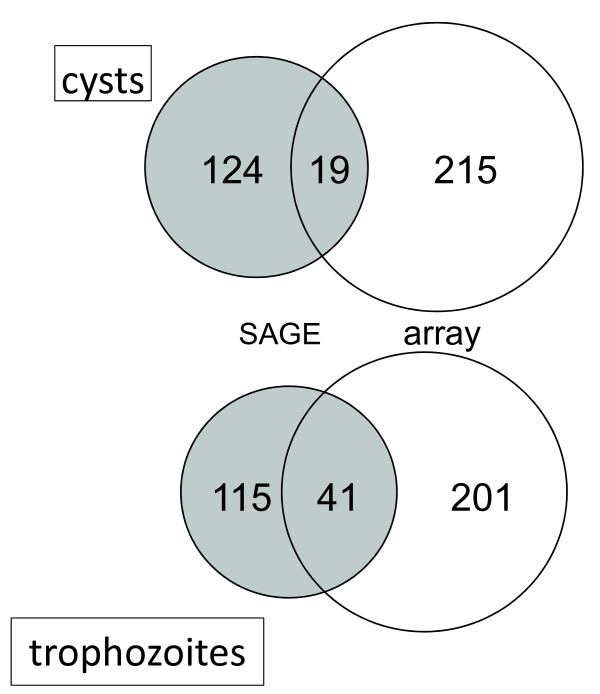
**Venn diagram of the number of highly expressed transcripts in SAGE and microarray analyses**. Genes representing ≥0.1% of SAGE tags were included. Areas in each diagram are proportional to the number of genes. Grey, SAGE [9]; white, microarray data from this study. Cyst microarray data originate from the analysis of cysts of isolate H3, whereas trophozoite microarray data are from WB isolate. Similar results were obtained with GS trophozoites (see text).

We compared our microarray data with the first comprehensive analysis of the *G. lamblia *transcriptome which was performed using SAGE [9]. Comparing SAGE and microarray data from cysts showed little correlation. For this comparison we included the 124 genes with 0.1% or more SAGE tags in cyst, and compared this list to 215 genes (see Additional file 2) with a mean (n = 6) cyst microarray fluorescence above background (Figure 3). This comparison revealed 19 matches, equivalent to only 15% (19/124) of the genes with at least 0.1% of SAGE tags. As an illustration, ubiquitin, one of our top-ranking genes was not represented among the cyst SAGE tags, and histone H4, which ranks second in our classification, was not detected either. Somewhat better correlated was the expression of histone H2B (microarray rank 3, SAGE rank 37) and dynein light chain (microarray 4^th^, SAGE 26th). The overall lack of correlation between cyst datasets could have several reasons, including experimental differences between the two studies. The fact that the cysts used in our study were obtained from gerbils, whereas Birkeland and colleagues produced cysts in vitro [18], was considered as a possible cause of the poor correlation between cyst datasets. To investigate this possibility, we compared SAGE and microarray datasets from trophozoites (Figure 3). Because the culture conditions used in both studies were similar, one would expect to find a better overlap than observed with cysts. As for the comparison of the cyst data, we considered genes contributing at least 0.1% of trophozoite SAGE tags (n = 115, 3.8% of detected genes) and 201 genes with the highest microarray fluorescence value. By including 201 genes from the microarray data, the ratio of SAGE/microarray genes is the same for the cyst and trophozoite comparison (1:1.75). Indeed, in the trophozoite data comparison 36% (41/115) of SAGE genes were present in the microarray gene list. To ensure that the use of assemblage B cysts and assemblage A trophozoites did not affect these results, the SAGE-microarray comparison was repeated with two replicate microarray datasets originating from GS (assemblage B) trophozoites. This analysis gave similar results with 31% (36/115) genes shared by the microarray and SAGE trophozoite datasets. Thus the percentage of matches among trophozoite datasets was about twice that found in the cyst comparison. This observation raises the possibility that cysts produced in vitro and cysts originating from an infection express a different set of genes.

### Expression of histone and histone modifying enzymes

The high level of histone mRNA in cysts raises indicates the importance of histone metabolism in cyst. To gain further insights into this function we compared the expression of core histones and histone modifying enzymes in trophozoites and cysts. Table 4 shows that core histones were expressed in both life cycle stages, whereas histone modifying enzymes were only expressed in trophozoites.

**Table 4:** Histone and histone modification enzymes*

	microarray					
			
	trophozoites (n = 3)		cysts (n = 6)		annotation	gene_ID
			
	mean	SD†	mean	SD		
core histones	184,230	76,664	87,271	66,049	Histone H3	GL50803_3367
	311,567	256,317	477,105	254,294	Histone H3	GL50803_135231
	762,551	688,884	1,577,324	1,090,907	Histone H2B	GL50803_121046
	1,475,752	1,596,837	2,057,058	1,376,160	Histone H4	GL50803_135002
	571,946	4,134,038	176,800	144,668	Histone H2A	GL50803_14256

acetylases	49,223	12,186	0		Histone acetyltransf. MYST2	GL50803_2851
	124,837	63,033	0		Histone acetyltransf. B sub. 2	GL50803_14753

methylases	34,033	42,382	0		Set-2, putative	GL50803_8921
	11,028	19,092	0		hypothetical protein^#^	GL50803_13838
	57,178	37,638	0		hypothetical protein^#^	GL50803_13790
	95,539	31,724	0		hypothetical protein^#^	GL50803_17036

deacetylase	16,367	25,657	0		Histone deacetylase	GL50803_3281

*histones and modifying enzymes not detected on microarrays are not shown

^†^standard deviation

^#^annotated as methylases by Sonda et al. (2010) [23]

## Discussion

The fact that the entire life cycle of *G. lamblia *can be reproduced in vitro makes this species an attractive model to study the differentiation of cyst into trophozoite and the reverse process of encystation. Recently, genome-wide studies of *G. lamblia *transcriptional regulation have been undertaken [9,12] but no global comparison of the cyst and trophozoite transcriptome has to our knowledge been published. The study of the trophozoite and cyst transcriptome is relevant to understanding the *G. lamblia *life cycle and the evolution of encysted forms which are essential to the survival of many enteric organisms. Given that cysts don't divide and are assumed to have little metabolic activity, it is likely that for many proteins in cysts no mRNA is present. Combined transcriptome and proteome analyses [7] will generate a more comprehensive view of the composition and metabolic activity of cysts.

Microarray and RT PCR data clearly show that the cyst transcriptome is much reduced in terms of abundance and complexity as compared to that of trophozoites. DAVID analysis of over-represented GO terms [19] suggests an overall resemblance in the composition of the transcriptome throughout the life cycle, but the analysis of highly expressed genes highlights significant differences.

As in most quantitative analyses, the comparison of microarray data required calibration against a benchmark. As described in Methods below, we used RNA quantity of as benchmark by using an equal amount of amplified RNA for preparing Cy3 labelled probes. The differences in transcript levels are thus to be interpreted as relative to total RNA extracted from cysts and trophozoites.

To what extent rRNA and tRNA which constitutes the bulk of cellular RNA varies is unknown. An alternative calibration would have been to normalize the data against the number of cysts, trophozoites or nuclei. This approach was discarded because of the possibility that extraction of RNA from cysts is less efficient than extraction from trophozoites. Had we chosen to normalize against cell number, it would have been difficult to assess whether differences between cyst and trophozoite were genuine or a result of cyst nucleic acid being more difficult to extract.

The experiments were constrained by the fact that *G. lamblia *microarrays are designed from the assemblage A genome and that the only source of cysts we could identify uses assemblage B. Because DNA sequence identity between assemblage A and B genome averages 77% [3], the possibility that analyzing assemblage B cyst cDNA with assemblage A microarrays could artificially reduce the hybridization signal was considered. Replicate microarray hybridizations were performed with cDNA originating from assemblage A and B trophozoites (Additional file 1). These controls showed no evidence of differential hybridization of cDNA originating from different assemblages under the hybridization conditions we used. This does not exclude that highly polymorphic transcripts were missed, but indicates that for the vast majority of genes annealing to the 70 mer microarray oligonucleotides was sufficiently stable to tolerate mismatches. Moreover, the vast majority of fluorescent signal from *Arabidopsis *control spots and empty spots present on the array were well below background (mean Cy3 fluorescence = 1552, n = 3860), confirming the specificity of the hybridization signal and demonstrating adequate stringency of the hybridization protocol.

Because we expected significant differences in the magnitude and diversity of cyst and trophozoite mRNA transcriptome we did not directly compare trophozoite and cyst transcriptome using a conventional 2-color microarray protocol. Two-color microarrays require normalization to eliminate the effect of differential labelling of dyes, which is typically accomplished with microarray analysis programs [20]. These programs normalize Cy3 and Cy5 fluorescence based on the assumption that the samples being compared contain similar amounts of mRNA, as would be the cases with, say, healthy and diseased cells. Since we did not expect this assumption to hold, we chose to use only background-subtracted single-channel Cy3 fluorescence values. Since these data originated from calibrated amounts of Cy3 labelled probe, the resulting data are directly comparable. In the context of this study, an additional advantage of the single-dye design over a more conventional Cy3/Cy5 ratio is the feasibility to include fluorescence values below background, i.e., values equal zero. Since a large proportion of transcripts were not detected in cysts, the exclusion of ratios with a numerator or denominator equal zero would have excluded biologically relevant information.

The elevated expression of some genes observed in the microarray dataset confirms previous observations. For instance, we found high levels of ubiquitin mRNA in trophozoites and cysts, which is consistent with previous RT PCR analyses [21]. The expression of ubiquitin in trophozoites is not unexpected, but the abundance of ubiquitin mRNA in cysts suggests extensive protein turn-over. Other top-ranking genes in cysts and trophozoites include histone. This observation is consistent with the constitutive expression of various histones during the trophozoite mitotic cycle [22], but had not been observed previously in cysts. The absence of mRNA encoding histone modifying enzymes suggests that histone modification does not occur in cysts, and is consistent with many genes not being transcribed in this phase of the life cycle. This interpretation is in agreement with the previously observed decrease of histone acetylation during trophozoite encystation and the predicted importance of epigenetic regulation of transcription in the life cycle of *G. lamblia *[23]. Finally, we notice the unexpected expression in cysts of several genes encoding variant surface protein.

The comparison of SAGE and microarray data raises interesting questions regarding the properties of cysts produced in culture. Cysts encysted in vitro have been extensively characterized with respect to morphology, antigenic property [24], and cyst wall biosynthesis [25], as have many processes occurring during encystation. A direct comparison of the transcriptome and proteome of native cysts and cyst produced in vitro has to our knowledge not been performed. In light of the results presented here, such an analysis is warranted to assess to what extent cysts produced in vitro can serve as surrogates for native cysts. As RNA-Seq has become a more widely available technique for transcriptome profiling, an accurate comparison of the cyst transcriptome is now feasible.

## Conclusions

The transcriptome of *G. lamblia *cysts and trophozoites was investigated using oligonucleotide microarrays. Although in both life cycle stages transcripts related to ribosomal function are overrepresented, clear quantitative differences were observed. This global comparison of the cyst and trophozoite transcriptome indicates that, in comparison to trophozoites, in cysts only about 5% of mRNA species are expressed at level detectable with microarrays.

## Methods

### *G. lamblia *cysts and trophozoites

*G. lamblia *cysts of assemblage B isolate H3 from experimentally infected gerbils were purchased from Waterborne (New Orleans, Louisiana). Cyst viability was assessed by monitoring exclusion of propidium iodide as described [17]. Cysts were processed for RNA extraction within five days of shedding. Trophozoites of assemblage A isolate WB and assemblage B isolate GS were cultured in TYI-S-33 medium [26]. Trophozoites grown for 24 h or 72 h were counted with a hemocytometer, pelleted by centrifugation and washed in PBS prior to RNA extraction.

### RNA extraction, amplification and microarrays

Total RNA for microarray analysis was isolated using Trizol from trophozoites and cysts following 5 cycles of freeze/thawing. DNA was removed using the TurboDNase kit from Applied Biosystems/Ambion (Austin, Texas) and the RNA extracted with Qiagen RNeasy columns (Qiagen, Valencia, California) according to the RNA cleanup protocol. RNA quality was checked by running a portion of selected samples on an agarose gel and measuring absorbance at 260 nm and 280 nm. RNA was amplified in vitro with the WT-Ovation Pico RNA Amplification System (NuGEN, San Carlos, California). For the amplification reaction up to 5 μl of total RNA sample (50 ng) was used as substrate. A total of 2 μg cDNA was labelled using a Genomic DNA Enzymatic Labeling Kit from Agilent (Santa Clara, California).

Oligonucleotide microarrays were provided by the National Institutes of Allergy and Infectious Diseases (NIAID) Pathogen Functional Genomics Research Center. The arrays (*Giardia lamblia *microarray version 2) contain 19,230 elements consisting of duplicates of 70 mer oligomers derived from 9,115 predicted open-reading frames (ORFs) including the clearly indentified 6,470 ORFs of the genome of *G. lamblia *WB C6 (assemblage A). Also spotted on the slides are 500 *Arabidopsis thaliana *control oligomers. To prehybridize, slides were placed in a coplin jar containing 50 ml preheated prehybridization buffer (20× SSC, 10% SDS, 0.5 g BSA) and incubated at 42°C for 2 hr. Slides were then washed using filtered distilled water and isopropyl alcohol for 2 m and dried by centrifugation. To perform hybridization, labeled cDNA was dissolved in 50 μl of hybridization buffer (40% formamide, 5× SSC, 0.1% SDS, 0.1 M DTT). In some experiments 2 μl of universal microarray standard set was added to the probe mixture, and the probe denatured for 10 min at 95°C. a volume of 50 μl of probe was added to microarray slide and covered with LifterSlip coverslips (Erie Scientific, Portsmouth, New Hampshire). Slides were incubated in a 42°C water bath for 16-20 h. For post-hybridization wash slides were first submerged into a low stringency solution (2 × SSC, 0.1% SDS) preheated to 55°C and washed twice for 5 min each on a shaker. Slides were subsequently washed twice in medium stringency solution (0.1× SSC, 0.1% SDS), followed by two more 5-min washes at high stringency (0.1× SSC) at room temperature. Slides were dried in a centrifuge and scanned in an Agilent scanner.

### Data analysis

Files in TIFF format generated by the scanner were imported into TIGR_Spotfinder software [27]. Spots were manually curated to exclude artifactual spots and background cut-off was set at 5%. Cy3 fluorescence values output by Spotfinder were exported to Microsoft Excel. Fluorescence values from duplicate spots were averaged and the mean over six cyst biological replicates determined. Each cyst expression value used in the analyses is thus based on 12 individual fluorescence reading. For trophozoites, two microarray hybridizations were performed with GS trophozoites and three with WB trophozoites, for a total of four and eight fluorescence readings per gene. The DAVID suite of bioinformatics tools was used to identify functional annotations which are enriched as compared to the *G. lamblia *genome annotation. The program was accessed through the web interface at http://david.abcc.ncifcrf.gov/tools.jsp.

### RT- PCR validation

cDNA amplified in vitro as described above was diluted 100-fold and 1 μl of this dilution was amplified by PCR. PCR was performed in 20-μl capillary tubes using a LightCycler (Roche Diagnostics, Indianapolis, Indiana) thermal cycler. Reaction mixtures contained 1× LC-Fast Start DNA master mix for SYBR Green I (Roche Diagnostics), 3 mM MgCl_2_, 20 pmol each of forward and reverse primers, and 1 μl of cDNA template. The primer sequences are shown in Table 2. The PCR program included a denaturation step of 10 min at 95°C followed by 45 cycles of 1 s at 95°C, annealing for 8-9 s, and a 8-s extension at 72°C. Following amplification, the PCR products were subjected to melting curve analysis by raising the temperature from 45 to 95°C at a rate of 0.05°C/s. During the initial optimization phase PCR products were also electrophoresed on agarose gels to ensure that products of the correct size were amplified. Because trophozoites and cysts originated from assemblage A and B, respectively, we verified that the PCR results were not affected by the genotype. Equivalent amounts of DNA from assemblage A isolate WB and assemblage B isolate GS were amplified in parallel using primers specific for portion of the ubiquitin, histone H2B and 14-3-3 protein shown in Table 2. No systematic bias that could be linked to the genotype was observed.

## Authors' contributions

The study was designed by GW and ZF. ZF performed the experiments. ZF and GW analyzed the data. GW performed the statistical analysis. The manuscript was written by GW and ZF. Both authors approved the final manuscript.

## Disclaimer

The comments and views detailed herein may not necessarily reflect the views of the WateReuse Research Foundation, its officers, directors, employees, affiliates or agents.

## Data deposition

Microarray data were deposited in the GEO database [GPL:11228].

## Supplementary Material

Additional file 1 **Comparison of Cy3 fluorescence emitted by microarrays hybridized with assemblage A and B trophozoite cDNA**. Fluorescence values are means of two replicate microarray spots and are ranked in order of decreasing intensity, as in Figure 1. All datasets are biologically independent; the 3-digit microarray number is shown in the legend. Fluorescence and rank are plotted on a log scale. Isolate WB (red lines) is assemblage A, isolate GS (green line) assemblage B. Click here for file

Additional file 2 **Gene ID of 215 cyst and trophozoite genes which generated the highest mean Cy3 fluorescence**. Microsoft Excel file Click here for file
