# Supplementary figures and images for: A comparison of the Giardia lamblia trophozoite and cyst transcriptome using microarrays
Source: BMC Microbiol. 2011 May 4;11:91. doi: 10.1186/1471-2180-11-91 (PMC3096902; doi:10.1186/1471-2180-11-91)

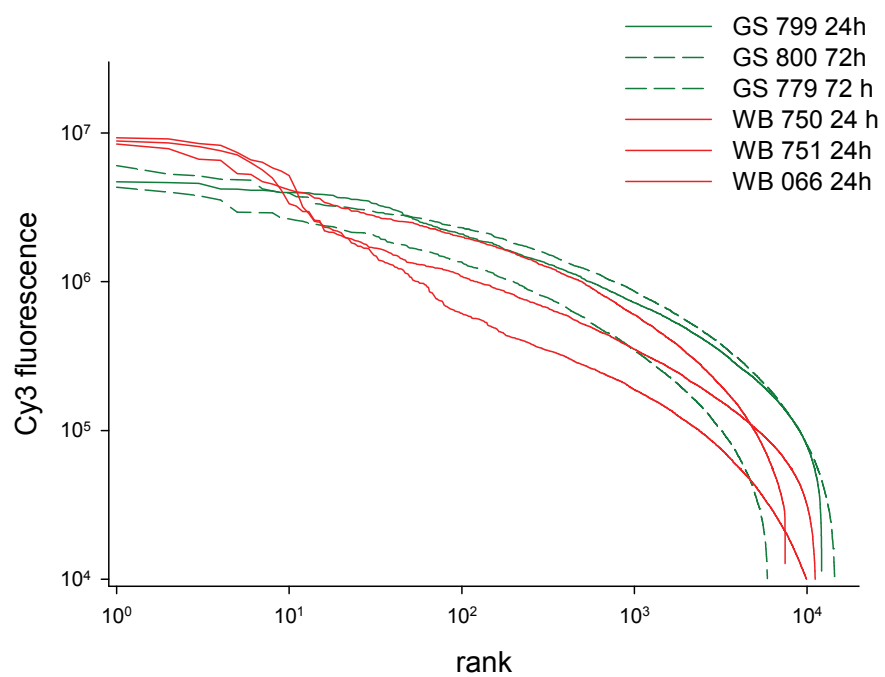

Supplement: Additional file 1 — Comparison of Cy3 fluorescence emitted by microarrays hybridized with assemblage A and B trophozoite cDNA. Fluorescence values are means of two replicate microarray spots and are ranked in order of decreasing intensity, as in Figure 1. All datasets are biologically independent; the 3-digit microarray number is shown in the legend. Fluorescence and rank are plotted on a log scale. Isolate WB (red lines) is assemblage A, isolate GS (green line) assemblage B. [file 1471-2180-11-91-S1.PDF]
